# Endotracheal tube microbiome in hospitalized patients defined largely by hospital environment

**DOI:** 10.1186/s12931-022-02086-7

**Published:** 2022-06-24

**Authors:** Erika Alejandra Cifuentes, Maria A. Sierra, Andrés Felipe Yepes, Ana Margarita Baldión, José Antonio Rojas, Carlos Arturo Álvarez-Moreno, Juan Manuel Anzola, María Mercedes Zambrano, Monica G. Huertas

**Affiliations:** 1 Corporación CorpoGen Research Center, Bogotá, Colombia; 2 Tri-Institutional Computational Biology & Medicine Program, Weill Cornell Medicine, New York, NY USA; 3 Hospital Universitario Fundación Santa Fe de Bogotá, Bogotá, Colombia; 4 Clínica Universitaria Colombia, Clínica Colsanitas, Bogotá, Colombia; 5 Universidad Central, Bogotá, Colombia; 6 Universidad Pedagógica y Tecnológica de Colombia, Tunja, Boyacá, Colombia

**Keywords:** Endotracheal tubes, Respiratory tract microbiome, Intensive care units (ICUs), Ventilator-associated pneumonia, Microbial diversity

## Abstract

**Background:**

Studies of the respiratory tract microbiome primarily focus on airway and lung microbial diversity, but it is still unclear how these microbial communities may be affected by intubation and long periods in intensive care units (ICU), an aspect that today could aid in the understanding of COVID19 progression and disease severity. This study aimed to explore and characterize the endotracheal tube (ETT) microbiome by analyzing ETT-associated microbial communities.

**Methods:**

This descriptive study was carried out on adult patients subjected to invasive mechanical ventilation from 2 to 21 days. ETT samples were obtained from 115 patients from ICU units in two hospitals. Bacteria isolated from endotracheal tubes belonging to the ESKAPE group were analyzed for biofilm formation using crystal violet quantification. Microbial profiles were obtained using Illumina sequencing of 16S rRNA gene.

**Results:**

The ETT microbiome was mainly composed by the phyla Proteobacteria, Firmicutes and Bacteroidetes. Microbiome composition correlated with the ICU in which patients were hospitalized, while intubation time and diagnosis of ventilator-associated pneumonia (VAP) did not show any significant association.

**Conclusion:**

These results suggest that the ICU environment, or medical practices, could be a key to microbial colonization and have a direct influence on the ETT microbiomes of patients that require mechanical ventilation.

**Supplementary Information:**

The online version contains supplementary material available at 10.1186/s12931-022-02086-7.

## Background

The lower respiratory tract harbors a selective microbial community composed mainly by bacteria of the phyla Bacteroidetes, Proteobacteria, Firmicutes, and the genera *Prevotella, Veillonella*, and *Streptococcus* [1, 2]. The microbiome of a healthy individual, according to the ecological modeling of the respiratory microbiome adapted from the island model of lung biogeography proposed by Dickson et al. [3], is thought to be determined by migration rate balance due to microaspiration, bacteria inhalation, and direct mucosal dispersion. Additional relevant factors for microbial colonization are elimination rates, which are determined by mucociliary clearance, coughing reflex and the host’s immune response [4]. The use of medical devices such as endotracheal tubes (ETTs) also increases the risk of microbial proliferation, primarily when used for long periods of time. Moreover, ETTs have been shown to promote lower respiratory tract colonization by oral microbial taxa, a process that could result in host immune system suppression and infection [5].

ETTs are quickly colonized by biofilm-forming bacteria [6], which have been reported to be better than planktonic cells at surviving environmental stress, antimicrobial therapy and immune system responses [7]. It has been estimated that biofilms may be implicated in approximately 65% of all infections, including those associated with the use of medical devices, which are strongly related to higher rates of morbidity and mortality [8]. These infections include ventilator-associated pneumonia (VAP), central line-associated bloodstream infection and catheter-associated urinary tract infection [9]. Most of these infections are caused by ESKAPE bacteria (*Enterococcus faecium, Staphylococcus aureus, Klebsiella pneumoniae, Acinetobacter baumannii, Pseudomonas aeruginosa* and *Enterobacter* spp), a group of pathogens that is also recognized for their ability to develop resistance to antibiotics, which undermines treatment and increases the rates of morbidity and mortality [10].

Prolonged ICU stays have been described as the topmost risk factor for healthcare-associated infections (HAIs), followed by invasive procedures, immunosuppressive treatments, parenteral nutrition, and blood transfusion [11]. Despite the availability of several methods for preventing HAIs in ICUs, such as surface disinfection, it is estimated that almost 40% of infectious microorganisms are carried and transmitted by healthcare workers and invasive procedures [11].

In order to identify HAI-related microorganisms, culture-dependent methods are commonly used [12]. These methods usually result in the isolation of specific microorganisms that do not necessarily reflect the entire microbial composition of a given sample. In this respect, high-throughput culture-independent approaches can be used to probe communities, obtain taxonomic profiles, and generate a more comprehensive view of a community. For example, a metagenomic study of the human lungs revealed colonization of the lungs by multiple microorganisms, even in healthy individuals [4].

In Colombia, there is a lack of information regarding lung microbial communities and their variations due to medical-related conditions or the use of ETTs. Only one study reported using 16S rRNA gene sequence analysis to identify oropharynx microbial communities in patients with pulmonary tuberculosis [13]. Considering the increasing number of ICU patients that require ETTs and mechanical ventilation during the COVID19 pandemic, it is of utmost importance to explore and expand our knowledge of these microbial communities and their possible impact on disease outcome. We hereby study the ETT-associated microbial communities obtained from ICU patients that required mechanical ventilation, with the aim of understanding if these communities showed an association with patient clinical variables.

## Methods

### Study design and sample collection

**Fig. 1:**
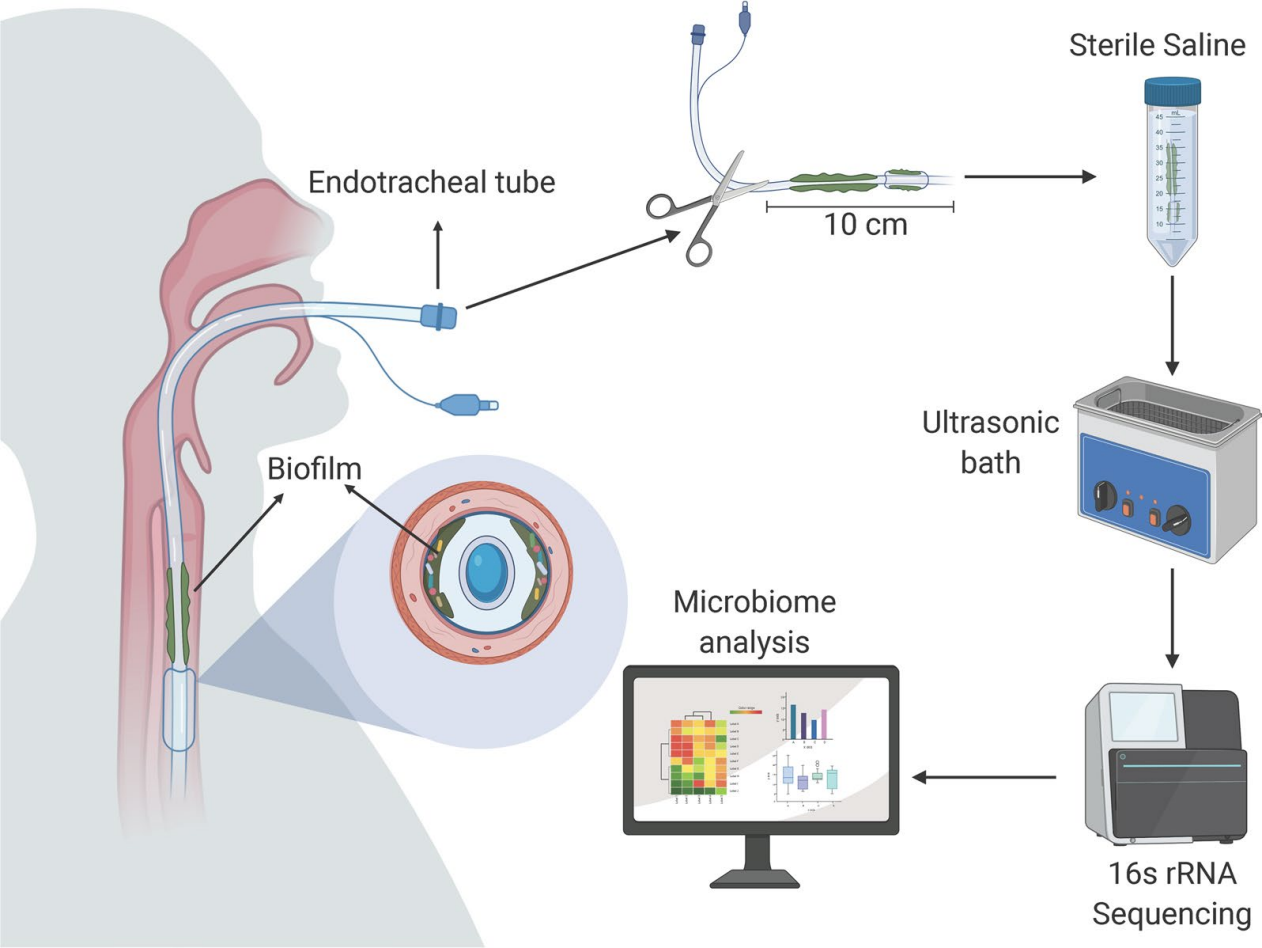
Sampling strategy. ETT samples were collected and used for both analysis of microbial communities by sequencing of the 16S rRNA gene and for culturing in laboratory media. A section of the endotracheal tube was cut and immediately placed in 0.85% NaCl. Associated bacteria were dislodged by three cycles of vortex followed by sonication. DNA was extracted and the V3-V4 hypervariable region of the 16S rRNA gene was amplified and sequenced on the Illumina MiSeq platform

To analyze the effect of ETT intubation on patients with mechanical ventilation, we obtained ETT samples from ICU (different facilities) patients in two hospitals (Clínica Universitaria Colombia (ICU-1) and Hospital Fundación Santa Fe (ICU-2)) in Bogotá, Colombia, between December 2016 and December 2017. Only patients who required ETT ventilation for more than 48 h were included in this study (n = 115), and all samples were obtained from adult patients that received mechanical ventilation between 2 to 21 days. The sampling strategy used for this study is shown in Fig. 1. Collected data included: patient variables (sex, age), medical background (oncological background, transplant, use of steroids, VAP, previous antibiotic use, previous hospital stay, use of antibiotic during intubation), and endotracheal intubation length of time. Ethical approval for ETT collection was obtained from the ethical committee of each of the corresponding hospitals. Given that no further procedures were needed, informed consent was not considered necessary.

### Sample processing and culture isolation

ETTs were removed (extubation) by medical staff, immediately placed in 500 mL sterile glass bottles (Schott, Germany), and processed at the Microbiology Laboratory of the University Hospital Fundación Santa Fe. 10 cm from the distal part of each tube were cut and placed in 0.85% NaCl. Associated bacteria were dislodged using three vortex cycles (30 s) followed by sonication (60 s).

The suspension obtained from each ETT was used to recover microorganisms on blood agar (bioMérieux, France), MacConkey agar (bioMérieux, France) and Chocolate agar (bioMérieux, France), incubating at 37 °C for 18–24 h [14]. Bacterial identification was carried out employing the VITEK® 2 automated system (bioMérieux, France). All samples were handled by the same person, stored at 4 °C for 2–4 h prior to further analyses, taking care to minimize variability associated with sample handling.

### DNA extraction and gene sequencing

DNA was extracted from the suspension using the Powersoil Kit (Qiagen), and the V3-V4 hypervariable region of the 16S rRNA gene was amplified using 341F and 805R primers [15] with the following conditions: 95 °C for 3 min, followed by 35 cycles of 20 s at 95 °C, 20 s at 52 °C, 60 s at 65 °C, and finally, 6 min at 72 °C. Each 20 μL PCR reaction was prepared with 4 μL 5 × HOT FIREPol master mix (Solis BioDyne, Tartu, Estonia), 2 μL of each primer (10 μM), 2 μL of sample DNA and 12 μL PCR-grade water. Negative controls were carried out for all PCR reactions. The amplicons were pooled in equimolar concentrations using the SequalPrep plate normalization kit (Invitrogen, Carlsbad, CA, United States) and then purified with AMPure XP beads (Beckman Coulter, Atlanta, GA, United States). Amplicons were sequenced on the Illumina MiSeq platform with paired-end reads (2 × 250 bp).

### Sequence processing, boinformatics and statistical analysis

Illumina reads were quality checked with FastQC [16] and edited with Trimmomatic [17] to remove the adapter and low-quality sequences that included reads with ambiguous nucleotides (q value < 25) and short reads (< 200 bp). Sequences were analyzed using the standard operating procedure from MOTHUR (v.1.39.5) [18], and clustered into operational taxonomic units (OTUs) using the nearest neighbor algorithm with a 97% similarity cut-off and classified based on the Greengenes database (v 13.8) [19]. Samples were normalized to 3934 sequences, as a customized cut-off for being one of the smallest numbers without losing too many patients that had lower number of sequences. After normalization, 110 samples were recovered and used for further analyses. 7,284,094 sequences longer than 200 bp were recovered.

Diversity within samples (alpha-diversity) was analyzed with the Shannon–Weaver [20] and Simpson indexes [21]. Richness of microbial communities was assessed based on the observed number of OTUs, Chao1, and the rarefaction curves using the R package Phyloseq [22]. Multiple comparisons of richness and diversity measures were performed by one-way ANOVA and Welch's t-test, with *P* values of < 0.05 considered statistically significant. Microbial community comparisons (beta-diversity) were assessed with a Principal Coordinate Analysis (PCoA) based on the Bray–Curtis index matrix. Significant differences were computed by performing a Permutational Multivariate Analysis of Variance Using Distance Matrices (Adonis in R). *P* values were considered significant with *p* < 0.05. We used ALDEx2 analysis (ANOVA-Like Differential Expression tool for compositional data) [23] to find OTUs that define the differences between microbiomes. The ALDEx2 R package decomposes sample-to-sample variation into four parts (within-condition variation, between-condition variation, sampling variation, and general unexplained error) using Monte-Carlo sampling from a Dirichlet distribution (aldex.clr: denom = “all”). The statistical significance of each OTUs was determined by the Welch's t-test and Wilcoxon Test. The significant difference of the abundant OTUs was used to generate a heatmap based on the Bray–Curtis index. Abundances were clr (center log ratio) transformed.

To compare the hospital antibiotic use between the ICUs the Chi square test was used. It was considered statically significant at *p*-value < 0.05. We compared the differential abundances of bacteria upon antibiotics within clinics with ALDEx2 and identified differences with expected p-value of Welch's t-test and Wilcoxon rank test (p < 0.05). Only two classes of antibiotics (beta-lactams and glycopeptides) were tested, as they had patients in both antibiotics and non-antibiotics groups between the two clinics. Abundances of each significant bacteria were log-10 transformed.

### Evaluation of biofilm formation

The evaluation of biofilm formation was performed on the isolates belonging to the ESKAPE group from patients who developed VAP, according to previous protocols [24] and standardizations. A single bacterial colony was inoculated in 2 mL of liquid LB (Luria–Bertani) medium for *K. pneumoniae* and *P. aeruginosa* and 2 mL of liquid MH (Mueller–Hinton) medium for *S. aureus* and *E. faecium* under constant agitation of 180 rpm for 18 h. 1.5 µL of this pre-inoculum were placed into 150 µL of liquid MH in wells of 96-well polystyrene plates (Beckton Dickinson, USA). Plates were incubated 37 °C for 24 h. Wells were gently washed three times with distilled water, bacterial cells were fixed with methanol for 15 min and stained with violet crystal (0.1%) for 20 min. The wells were washed three times with distilled water and treated with SDS (10%) for 20 min to remove the stained cells. The absorbance was taken at 595 nm (OD595, Tecan, Switzerland). Medium without cells was used as negative control, and *K. pneumoniae* strain LM21 [24, 25] was used as positive control. For results interpretation, the cut-off point was established as the average of the OD: (OD of the negative control + (3 * Standard deviations) [26]. An isolate was considered to be a biofilm producer when its OD was higher than the OD of the cut-off point. Six essays were performed for each strain.

### Calculation of VAP incidence density

Incidence density was calculated as the percent of VAP cases within the study population. The VAP incidence density indicator was obtained using the following formula: (number of cases with VAP/number of ventilator days) × 1000 = VAP rate per 1000 ventilator days [27].

## Results

### High incidence of VAP in ICU-1

In this study we enrolled 115 adult patients that were intubated in two ICUs from two different hospitals and evaluated patient parameters and ETT-associated microbial communities. In this study 20% of the patients developed VAP (defined as pneumonia occurring 48 h after endotracheal intubation [28]). When the incidence density of VAP per 1000 ventilator days for both hospitals was calculated, we observed that VAP incidence density was higher in ICU-1 (29.9 per 1000 ventilator days) than in ICU-2 (21.7 per 1000 ventilator days).

### Microbial diversity shifts by clinic and days of intubation

**Fig. 2:**
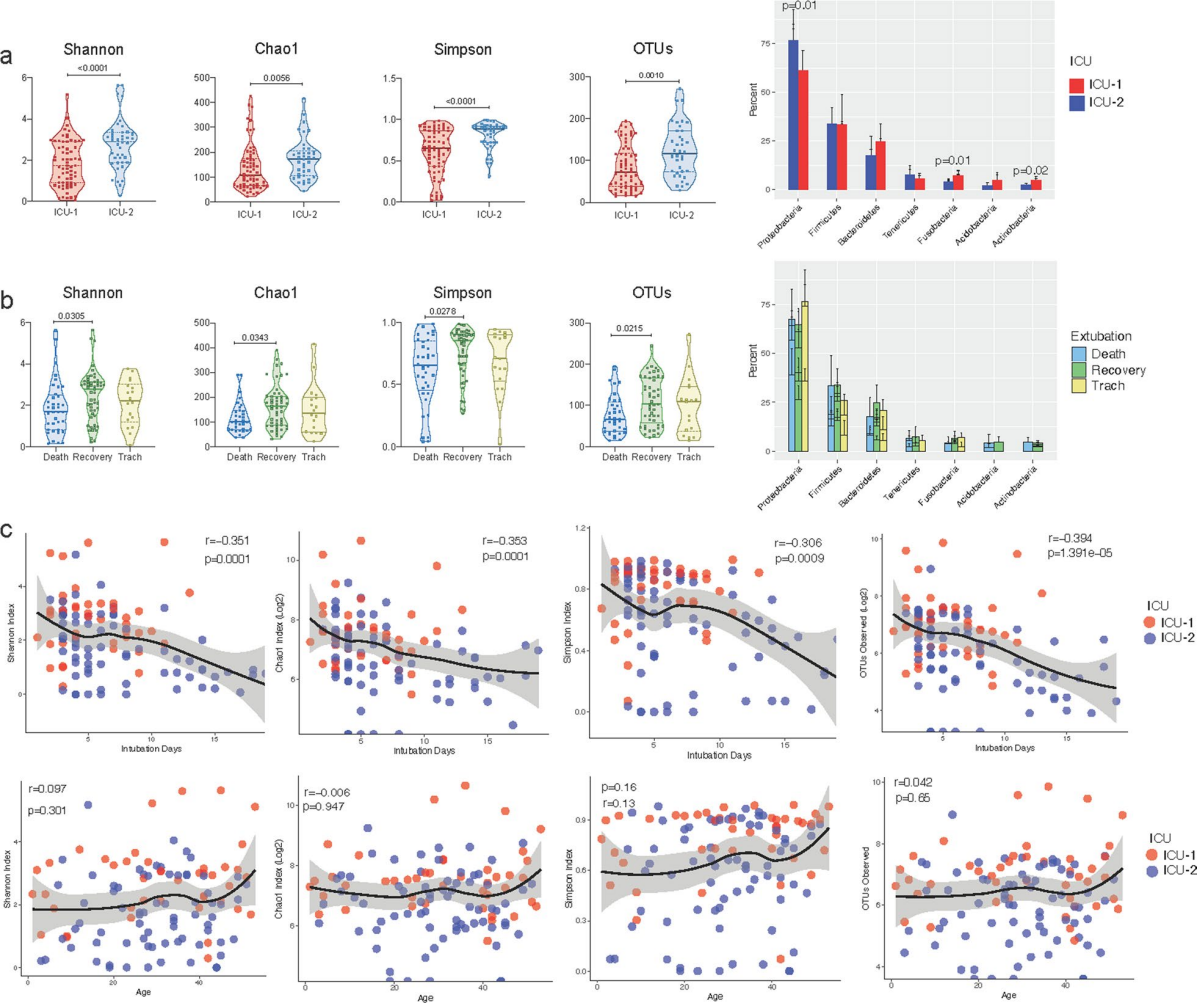
Alpha diversity comparisons. Violin plots showing community alpha diversity according to hospital (**a**) and extubation reason (**b**). Statistical differences based on ICU were assessed with Welch t-test (Shannon: *p* =  < 0.001, Chao1: *p* = 0.0056, Simpson *p* = 0.001, OTUs observed: *p* = 0.0010), and with One-way ANOVA for extubation reason (Shannon: *p* =  < 0.0305, Chao1: p = 0.0343, Simpson *p* = 0.0278, OTU number: *p* = 0.0215. **c** Intubation (top) and patient age (bottom) were tested for correlation using Spearman’s rank correlation coefficient (rho). Statistical differences were observed only for intubation days (Shannon: *p* =  < 0.001, Chao1: *p* = 0.0001, Simpson *p* = 0.0009, OTUs observed: *p* = 1.391e−05)

ETTs removed from patients, due to death, recovery, or tracheostomy, were used for analysis of microbial communities. Samples were processed and microbial community profiles were obtained by amplification and sequencing of the bacterial 16S rDNA V3-V4 hypervariable region using the MiSeq Illumina platform (see Fig. 1). A total of 6,352,541 sequences were obtained. Samples contained between 105 to 203,586 sequences with an average of 57,677 sequences per sample. After normalization to 3934 sequences, which retained 110 samples, rarefaction curves showed saturation for most samples (see Additional file 1: Fig. S1a). The number of observed OTUs, ranged from 15 to 927 OTUs per sample. Alpha diversity indexes were calculated for each ICU (Fig. 2a) (see Additional file 1, Fig. S1a), and statistical differences with Welch’s t student were observed (Shannon: *p* =  < 0.001, Chao1: *p* = 0.0056, Simpson *p* = 0.001, OTUs observed: *p* = 0.0010). For extubation reason one way ANOVA test was performed and we found statistical differences in the alpha diversity indexes (Shannon: *p* = 0.0305, Chao1: *p* = 0.0343, Simpson *p* = 0.0278, OTUs observed: *p* = 0.0215) (Fig. 2b). An analysis using ALDEx2 identified four OTUs (see Additional file 1: Fig. S2) that varied significantly among extubation reason, a finding that would have be further corroborated given the limited number of samples used here. The phyla Proteobacteria, Firmicutes, and Bacteroidetes were among the most abundant in both cases (ICUs and extubation reason). However, only the percent of Proteobacteria (p = 0.01), Fusobacteria (p = 0.01) and Actinobacteria (p = 0.02) showed significant differences in their abundance between ICUs (Fig. 2a).

To see if the microbial communities were influenced by other factors, the number of days that the patients remained under intubation (days of intubation) and patient age were also evaluated by correlation coefficients (Fig. 2c). Results indicate a moderate relationship (r value from − 0.301 to − 0.394) for all the alpha diversity indexes calculated, where microbial diversity decreases the longer a patient stays in intubation. In contrast, no relationship was found with the patient's age.

### ICU as a relevant factor

**Fig. 3:**
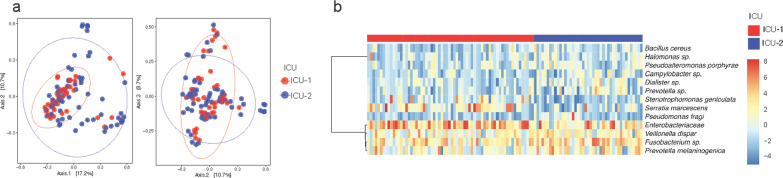
Sampling site defines composition. **a** PCoA axis 1:2 and 2:3 based on the Bray–Curtis index shows that patient microbiomes differ according to the hospital. Clusters were shown to be significant (*p* = 0.00199) calculated with ADONIS. **b** Heatmap of the 13 OTUs with significant differences in abundance found by ALDEx2

We analyzed if there were any differences in patient characteristics between hospitals with Chi square test and found a statistically significant difference in the use of antibiotics before (*p* = 0.024) and during intubation (*p* = 0.047) and in the use of beta-lactams (p < 0.001) (Table 1). We next analyzed if the microbiomes differed depending on the hospital in which a patient was hospitalized (Fig. 3). A PCoA showed that communities differed depending on the ICU (Fig. 3a). Patients from ICU-1 harbored more homogeneous microbial communities than those from ICU-2. These differences were significant (*p* = 0.0019) as determined by a Permutational Multivariate Analysis of Variance Using Distance Matrices (ADONIS), by first finding the centroids for each group and then calculating the squared deviations of each site to that centroid. To determine if specific taxa were driving this difference, we used ALDEx2 to identify OTUs that were significantly different in abundance between the two hospital ICUs. We identified 13 OTUs with significant differences as determined by the expected *p*-value of the Welch’s t test and the Wilcoxon rank test (Fig. 3b).

**Table 1:** Demographics and characteristics of the patients

	Location		
	ICU-1 (n = 73)	ICU-2 (n = 42)	
Age			
Mean ± SD	64 ± 14	67 ± 18,87	
Intubation days			
Median (quartile 1, quartile 3)	6 (4, 10)	4.5 (3, 8)	
Sex	n (%)	n (%)	
Male	43 (58.9)	19 (45.2)	
Female	30 (41.0)	23 (54.7)	
Population characteristics			
Oncological background	22 (30.1)	7 (16.66)	
Transplant	2 (2.73)	1 (2.38)	
Use of steroids	5 (6.84)	4 (9.52)	
Previous hospital stay	40 (54.7)	17 (40.47)	
VAP	18 (24.6)	5 (11.90)	
Extubation reason			
Recovery	28 (38.35)	28 (66.66)	
Death	36 (49.31)	6 (14.28)	
Tracheostomy	9 (12.32)	8 (19.04)	
Intubation length of stay (grouped by days)			
Long (> 16 days)	6 (8.22)	0 (0)	
Middle (6–15 days)	31 (42.46)	17 (40.47)	
Short (0–5 days)	36 (49.31)	25 (59.52)	
Hospital antibiotic use			p value*
Previous antibiotic use	45 (61.64)	16 (38.09)	0.024
Antibiotic during intubation time	63 (86.30)	29 (69.04)	0.047
Use of beta-lactams	58 (79.45)	22 (52.38)	< 0.001
Use of glycopeptides	11 (15.06)	7 (16.66)	0.80

Data is shown as number of patients and percentage. *Chi square test

An additional analysis was carried out comparing patients within clinics to investigate if antibiotics use modified abundance of some OTUs. This analysis was performed with beta-lactams (see Additional file 1: Fig. S3) since there were too few samples for other antibiotics (macrolides, aminoglycosides and quinolones). While in ICU-2 only one taxon showed significant differences with respect to beta-lactam use, 16 OTUs were shown to be affected in ICU-1. ICU-1 showed lower diversity and richness compared to ICU-2 (Fig. 2a), yet more differentially abundant bacteria were found within ICU-1, some of which belong to phyla that were in higher abundance in this clinic such as Bacteroidetes, Fusobacteria and Actinobacteria. For the use of glycopeptides, significant differences were found for 4 OTUs in ICU-2 (see Additional file 1: Fig. S4).

As can be seen in the heatmap, taxa from the Enterobacteriaceae family and the species *Serratia marcescens* were more abundant in ICU-1. In ICU-2 taxa corresponding to the genera *Prevotella, Dialister* and *Campylobacter* were more abundant. A PCoA performed based on reason for extubation (death, recovery, or tracheostomy) and intubation length of stay grouped by days (short 2–5 days), middle (6–15 days) and long (> 16 days) showed that there were no differences among communities using these criteria (see Additional file 1: Fig. S1c).

### High prevalence of *K. pneumoniae* and *E. coli* biofilm producers among VAP patients

To study bacteria associated with ETTs from patients that developed VAP, we recovered microorganisms on standard laboratory culture media. 22 bacterial isolates were cultured from 20 samples that corresponded to patients that developed VAP and, of these, 14 belonged to the ESKAPE group of pathogens (Table 2). When evaluated for biofilm formation, 8 of these 14 isolates could produce a biofilm using our assay. Of these, *K. pneumoniae* (3/5) and *E. coli* (4/4) were the most frequent biofilm-forming strains (Table 2). It is also important to highlight that several yeasts were also recovered from this group of patients, specifically *C. albicans*, which was cultured in 8 of the 20 ETT samples (Table 3).

**Table 2:** Biofilm producing isolates of the ESKAPE group in VAP patients

Bacteria	Number of isolates	Biofilm producing isolates
*S. aureus*	1	0
*A. baumanii*	1	0
*P. aeruginosa*	3	1
*E. coli*	4	4
*K. pneumoniae*	5	3

**Table 3:** Results obtained from culture isolation and identification via 16S rDNA sequencing for patients with VAP

Sample ID	Isolates	High-throughput sequencing of 16S rDNA*
8-F	No growth	*Prevotella melaninogenica, Fusobacterium* spp*., Enterococcus* spp.*, Neisseria subflava*
74-C	*E. coli, K. pneumoniae*	*Enterobacteriaceae* spp*., Stenotrophomas geniculata, Escherichia coli, Lactobacillus zeae, Halomonas* spp.
72-C	*C. albicans, M. catarrhalis*	*Haemophilus influenzae, Enterobacteriaceae* spp.*, Prevotella melaninogenica, Granulicatella* spp*., Fusobacterium* spp*., Serratia marcescens*
64-C	*A. baumanii*	*Acinetobacter rhizosphaerae, Veillonella dispar, Fusobacterium* spp*., Prevotella* spp*.*
60-C	*E. coli, K. pneumoniae,*	*Escherichia coli, Enterobacteriaceae* spp*., Staphylococcus* spp*., Streptococcus* spp*., Streptococcus infantis, Neisseria subflava*
	*S. maltophilia*	
54-C	*E. coli*	*Pseudomonas* spp*., Escherichia coli, Mycoplasma* spp*.,Staphylococcus* spp*., Enterobacteriaceae* spp*.*
52-C	*P. aeruginosa,*	*Serratia marcescens, Pseudomonas* spp*., Morganella morganii, Providencia* spp*., Enterococcus* spp*.*
	*S. marcescens, C. albicans*	
48-C	No growth	*Haemophilus influenzae, Enterobacteriaceae* spp*., Prevotella melaninogenica, Fusobacterium* spp*., Staphylococcus* spp*.*
33-C	*C. albicans, C. glabrata*	
10-F	*C. albicans, C. tropicalis*	*Enterobacteriaceae* spp*., Streptococcus infantis, Sinobacteraceae* spp*., Rhodoplanes* spp*., Bacillus cereus*
11-C	*R. planticolla, Citrobacter koseri*	*P. aeruginosa, Citrobacter koseri, Proteus* spp*., E. coli, Trabulsiella farmeri*
	*P. aeruginosa*	
15-F	*C. albicans*	*Veillonella dispar, Enterobacteriaceae* spp*., Sinobacteraceae* spp*., Streptococcus infantis, Thermomonosporacea* spp*.*
19-C	*P. aeruginosa,*	*Stenotrophomonas geniculata, Pseudomonas spp., Enterobacteriaceae, Mycoplasma spp., Parvimonas* spp*.*
	*K. pneumoniae, Proteus mirabilis*	
19-F	*S. epidermidis*	*Haemophilus influenzae, Staphylococcus* spp*., Enterobacteriaceae* spp*., Pseudomonas* spp*., Fusobacterium* spp*.*
1-C	*K. pneumoniae*	*Enterobacteriaceae* spp*., Veillonella dispar, Actinomyces* spp*., Abiotrophia* spp*., Streptococcus* spp*.*
20-C	*K. pneumoniae*	*Enterobacteriaceae* spp*., Escherichia coli, Enterococcus* spp*., Haemophilus influenzae, Neisseria subflava*
24-C	*C. albicans*	*Staphylococcus* spp*., Mycoplasma* spp*., Stenotrophomonas geniculata, Haemophilus influenzae, Elizabethkingia meningoseptica*
24-F	*C. albicans, Candida glabrata*	*Veillonella dispar, Streptococcus infantis, Streptococcus anginosus, Tannerella* spp*., Paludibacter* spp*.*
25-C	*C. albicans*	*Mycoplasma* spp*., Prevotella melaningogenica, Atopobium vaginae, Veillonella dispar, Prevotella* spp*.*
30-C	*S. aureus, S. marcescens,*	*Staphylococcus* spp*., Serratia marcescens, Escherichia coli, Stenotrophomonas geniculata, Prevotella* spp*.*
	*E. coli*	

^*^Five most abundant OTUs. Samples labeled with letter C belongs to ICU-1, samples labeled with letter F belongs to ICU-2

### 16S rDNA analysis reveals additional microbial diversity in VAP patients

The microbial communities associated with ETTs from VAP patients were further compared by looking at the 5 most abundant OTUs obtained by sequence analysis in each sample. As can be seen in Table 3, sequence analysis corroborated in many cases the results obtained by culturing samples. In addition, we were able to identify bacteria from samples where no growth was obtained (8F, 48C) and where only yeasts were recovered. Taxonomic profiles obtained by sequencing also revealed microorganisms that can be difficult to culture, such as *Mycoplasma, Veillonella dispar*, and other anaerobes of difficult recovery by traditional aerobic cultivation (Table 3).

## Discussion

Studies of the microbiome and its association with lung diseases have provided significant insight on how distinct microbial communities can act as potential indicators of specific chronic and infectious diseases [29]. In this study, ETT microbial community structure from ICU patients was dominated by Proteobacteria, Bacteroidetes and Firmicutes, phyla that are commonly found in lower respiratory samples from ICU patients and have also been shown to have an inhibitory effect on taxa such as *P. aeruginosa* [30–32]. The abundance of these taxa could be due to colonization by oral cavity microorganisms, previously described as commensals in the oral microbiome and the first to come in contact with ETTs in the respiratory tract [33]. Previous studies have shown that dental plaque bacteria may also be found on ETTs, indicating a possible source of contamination [34, 35]. The composition of the ETT microbiome has been correlated with the patient prognosis where the presence of pathogens like *P. aeruginosa* are an important predictor of patient outcome [36]. In this study our analysis was based on the OTU approach rather than the use of amplicon sequence variants (ASVs), which is gaining momentum for microbiome studies [37]. However, the use of OTUs is still broadly used and yields comparable results [38, 39]. In addition, the availability of the data can allow future comparisons as new analytical tools are developed.

In contrast to microbiome studies conducted from other body parts, in this study we found a small number of observed OTUs (15–927). Similar results have been reported in other lung microbiome studies [40, 41]. Here, patients that recovered, and therefore had their ETT removed, had more observed OTUs than patients who died (Fig. 2b). Recent studies have shown an association between mortality rate and a decrease in abundance of bacterial taxa [42].

Among the ESKAPE pathogens cultured from VAP patient samples, the majority were *K. pneumoniae* (n = 5), *E. coli* (n = 4), and *P. aeruginosa* (n = 3), a finding that confirms the Colombian National Health Institute’s (INS) reports that these three pathogens are the most prevalent in HAIs in Colombia [43]. Most of the isolates (8 out of 12) were also capable of forming biofilms, consistent with previous findings demonstrating that nosocomial pathogens dominate in ETT biofilm [44]. It is also interesting to note that *S. marcescens*, a pathogen that can result in severe infections and sepsis [45], was found in two samples of one of the ICUs using both culture and metagenomic approaches (Table 3, Fig. 3b). Previous outbreak reports have demonstrated that poor ICU hygiene protocol adherence can promote HAI caused by this pathogen [46]. Additionally, a patient’s oral hygiene before ETT placement is a key factor for prevention of VAP caused by *S. marcescens,* due to an observed association between oral and tracheal samples from VAP patients and the presence of this microorganism [47].

In Colombia, there were 5141 VAP cases by the end of 2019, and the rate per 1000 ventilator days was 2.5, according to the INS [43]. In this study, VAP incidence was higher in ICU-1 (Table 1), which could be explained by the difference in diversity indexes between both hospitals (Fig. 2a). However, this hypothesis should be confirmed by additional studies. The statistically significant differences in microbial community diversity between the two hospitals evaluated in this study could be affected by local conditions, such as clinical practice and staff differences. Other studies have previously observed diversity discrepancies, even between two ICUs located in the same hospital [48].

Endotracheal intubation can alter natural lung defense mechanisms and the microbial environment by permanently connecting the oropharynx to the lower respiratory tract, disrupting the lung's ecosystem [44]. ETTs may also alter the abundance and composition of lung microbiota by harboring microorganisms that produce biofilms and become more tolerant to antibiotics and the immune system. Our analysis of the ETT microbiomes, indicated that prolonged intubation and the ICU itself could impact community composition, probably due to ETT manipulation by healthcare staff. The community composition, particularly the richness and abundance of different phyla, may play a crucial role in a patient’s outcome. Although these results suggest that microbiome alpha diversity, and perhaps some taxa, could be a prognosis indicator, further studies should be performed to verify these findings.

There are key aspects to consider with respect to the comparisons carried out in this study that may influence the observed differences between ICUs. First, it is known that the epidemiological profiles of general, surgical, neurocritical, and cardiovascular ICUs in each institution differ greatly [49, 50]. In this work, we collected samples from different ICU facilities within the same institution, which makes accurate comparisons difficult between the two hospitals studied. Furthermore, as seen in Table 1 there is a statistically significant difference (*p* = 0.024) in antibiotic use prior to ICU admission, which reflects a significantly higher previous disease burden in ICU-1 patients when compared to ICU-2 patients. Moreover, there was also a statistically significant difference found in the antibiotic use during intubation (*p* = 0.047), possible as the result of distinct antibiotic use policies between ICUs and hospitals, as well as the epidemiological differences mentioned above. The use of antibiotics, in particular beta-lactams which are included in the guidelines for the empirical treatment of VAP [28], also suggests that they may play a role in the differentiation of the microbiome between ICUs. More importantly, some of the differential OTUs identified in ICU-1 and a few in ICU-2 have been previously reported to be associated with VAP, such as *Haemophilus *spp*,* Dialister spp [51], *Streptococcus infantis*, and *Prevotella melaninogenica* [52]*.* These differences may also help explain why the rate of VAP was higher in ICU-1. Finally, it is also important to note that possible contaminants and small variations in protocols, materials and ICU environment could influence the ETT microbiome from these low biomass samples, particularly in the absence of extraction controls. However, all samples were processed for DNA extraction in the same facility by the same person using the same kit and reagents. Samples were also processed at various timepoints based on the dates of collection, in some cases from both ICUs simultaneously, which also reduces introduction of contaminants that could bias the results.

Due to these facts, it is important to be cautious when interpreting the observed differences in ETT microbiomes from both institutions. Although the observed differences could be related to the epidemiological profiles from the different ICUs sampled within the same hospital, and the distinct antibiotic use policies between institutions, these results would have to be further tested with a larger cohort of patients.

As the COVID-19 pandemic has expanded throughout the world, the number of patients requiring intubation has increased. The COVID-19 disease, an infection of the lungs that may lead to severe respiratory distress and organ failures, can potentially impact human health in the long term and also affect the human microbiome [53]. At present, several studies have explored the role of the microbiome in COVID-19 patients [53–55], and only a few have revealed a possible impact on COVID-19 disease severity and immune response related to alterations in microbial communities in the lung [55]. Studies with a reduced number of patients have shown an increase in pathogenic and commensal microorganisms, pointing to a possible dysbiosis observed in both COVID-19 patients and patients with community-acquired pneumonia [56]. Both fungal and bacterial infections have also been observed in deceased COVID-19 patients [57]. The relevance of the lung microbiome to host health cannot be overlooked as it can contribute to lung immunity and protect against infections [58]. Alterations of the microbiome may contribute to increased inflammation and result in a poorly regulated immune response [59, 60]. Although further studies are needed to establish a direct influence of the lung microbiome in the development of severe pneumonia and progression of diseases such as COVID-19, studies such as this one contributes to our understanding of the microbial communities and their possible relation to disease severity and outcomes in patients who require mechanical ventilation.

## Conclusions

The analysis of the microbial communities in intubated patients indicated that ETT microbiomes were diverse, but this diversity decreased with increased days of intubation. The microbial communities differed between the hospitals studied and diversity correlated with the hospital in which samples were taken rather than with patient age or disease prognosis. This study suggests that microbial differences were related to ICU epidemiological profiles and antibiotic administration or use policies in each institution. Although additional work is needed to confirm these correlations, this study provides insight into the possible factors that can influence the lung microbiome in hospitalized patients and opens the way for studying how changes in microbial community diversity can serve as potential indicators of patient health status and prognosis within a hospital.

## Supplementary Information

**Additional file 1:** **Figure S1. (a)** Rarefaction curves showing saturation for most samples from ICU-1 and ICU-2. **(b) **Alpha diversity indexes for the clinical characteristics collected. No statistical differences were observed according to Welch t-test, p > 0.05. Principal coordinate analysis for extubation reason (death, recovery or tracheostomy) **(c)** and days of intubation (long > 16 days, middle 6–15 days, and short 0–5 days) **(d) **do not display differences. **Figure S2.** Effects of extubation reason on bacterial differential abundances. 4 OTUs showed significant differences when comparing patients who died, recovered or had a tracheostomy. All the differences were significant according to the expected P value of Welch’s t-test and the expected P value of the Wilcoxon rank test. **Figure S3.** Effect of beta-lactam use on OTU abundance. Significant differences in OTU abundance based on beta-lactam use in **(a) **ICU-1 and ICU-2 **(b).** All the differences were significant according to the expected P value of Welch’s t-test and the expected P value of the Wilcoxon rank test. **Figure S4.** Effect of glycopeptide use on OTU abundance. The use of glycopeptides resulted in significantly different abundances of 4 OTUs in ICU-2. All the differences were significant according to the expected P value of Welch’s t-test and the expected P value of the Wilcoxon rank test.

## Data Availability

Sequence data is available under accession numbers PRJNA771670.

## Abbreviations

ETT: Endotracheal tube
VAP: Ventilator-associated pneumonia
ICU: Intensive care units
HAIs: Healthcare-associated infections
OTU: Operational Taxonomic Unit
