# Supplementary material for: Endotracheal tube microbiome in hospitalized patients defined largely by hospital environment
Source: Respir Res. 2022 Jun 24;23:168. doi: 10.1186/s12931-022-02086-7 (PMC9233342; doi:10.1186/s12931-022-02086-7)
Supplement: Supplementary file 1 — Additional file 1: Figure S1. (a) Rarefaction curves showing saturation for most samples from ICU-1 and ICU-2. (b) Alpha diversity indexes for the clinical characteristics collected. No statistical differences were observed according to Welch t-test, p > 0.05. Principal coordinate analysis for extubation reason (death, recovery or tracheostomy) (c) and days of intubation (long > 16 days, middle 6–15 days, and short 0–5 days) (d) do not display differences. Figure S2. Effects of extubation reason on bacterial differential abundances. 4 OTUs showed significant differences when comparing patients who died, recovered or had a tracheostomy. All the differences were significant according to the expected P value of Welch’s t-test and the expected P value of the Wilcoxon rank test. Figure S3. Effect of beta-lactam use on OTU abundance. Significant differences in OTU abundance based on beta-lactam use in (a) ICU-1 and ICU-2 (b). All the differences were significant according to the expected P value of Welch’s t-test and the expected P value of the Wilcoxon rank test. Figure S4. Effect of glycopeptide use on OTU abundance. The use of glycopeptides resulted in significantly different abundances of 4 OTUs in ICU-2. All the differences were significant according to the expected P value of Welch’s t-test and the expected P value of the Wilcoxon rank test. [file 12931_2022_2086_MOESM1_ESM.docx]

**Journal:** Respiratory Research – BioMed Central

**Title:** Endotracheal Tube Microbiome in Hospitalized Patients Defined Largely by Hospital Environment

**Authors:** Erika Alejandra Cifuentes^1^, María Alejandra Sierra^1,2^, Andrés Felipe Yepes^3^, Ana Margarita Baldión^3^, José Antonio Rojas^4^, Carlos Arturo Álvarez-Moreno^4^, Juan Manuel Anzola^1^, María Mercedes Zambrano^1^, Mónica G Huertas^1,5*^

*Corresponding autor: [mogahuertas@gmail.com](mailto:mogahuertas@gmail.com), monica.huertas01@uptc.edu.co

**Additional files 1**

**Supplementary Figures**


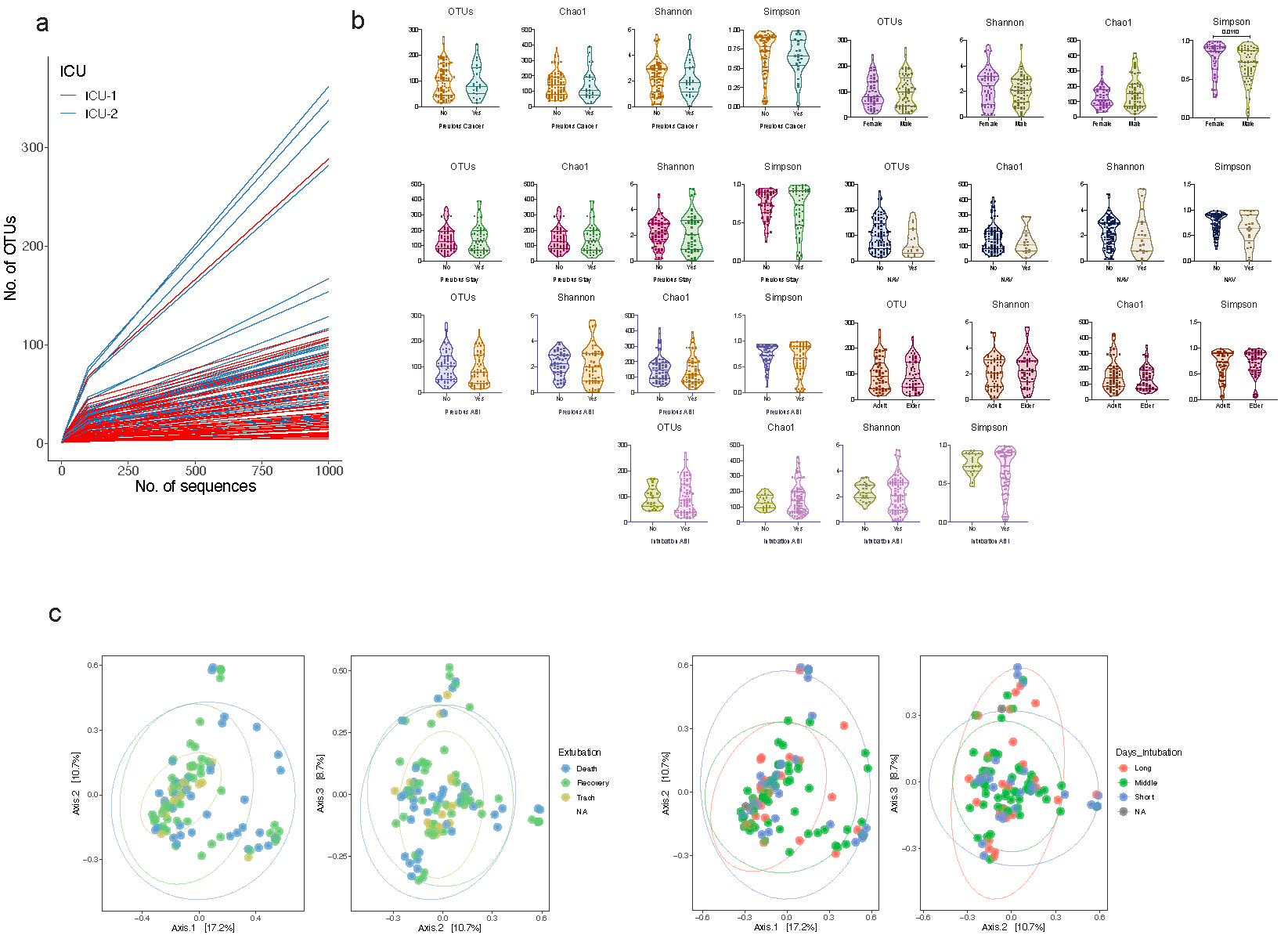


**Figure S1. (a)** Rarefaction curves showing saturation for most samples from ICU-1 and ICU-2. **(b)** Alpha diversity indexes for the clinical characteristics collected. No statistical differences were observed according to Welch t-test, p>0.05. Principal coordinate analysis for extubation reason (death, recovery or tracheostomy) **(c)** and days of intubation (long >16 days, middle 6-15 days, and short 0-5 days) **(d)** do not display differences.


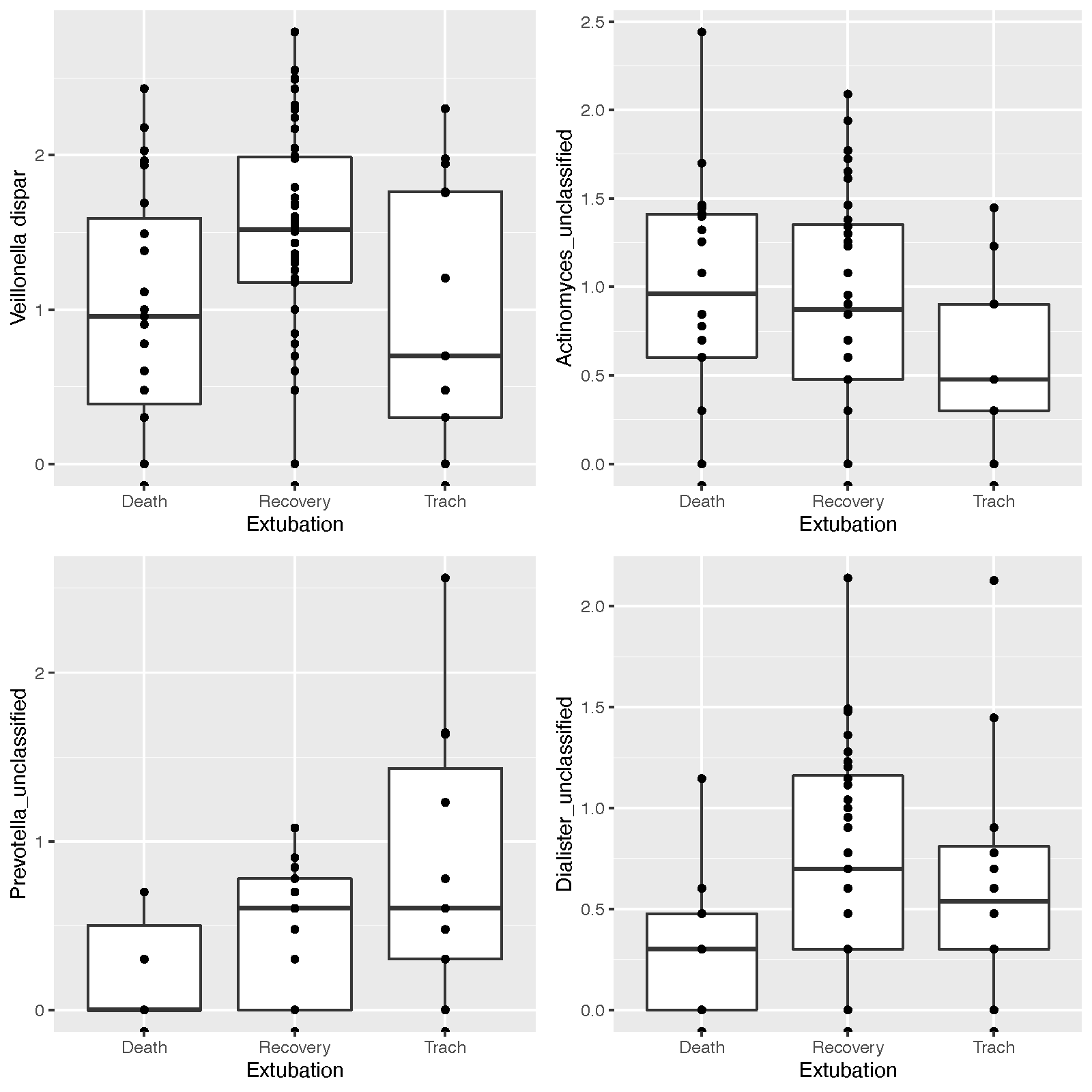


**Figure S2.** Effects of extubation reason on bacterial differential abundances.  4 OTUs showed significant differences when comparing patients who died, recovered or had a tracheostomy. All the differences were significant according to the expected P value of Welch’s t-test and the expected P value of the Wilcoxon rank test.


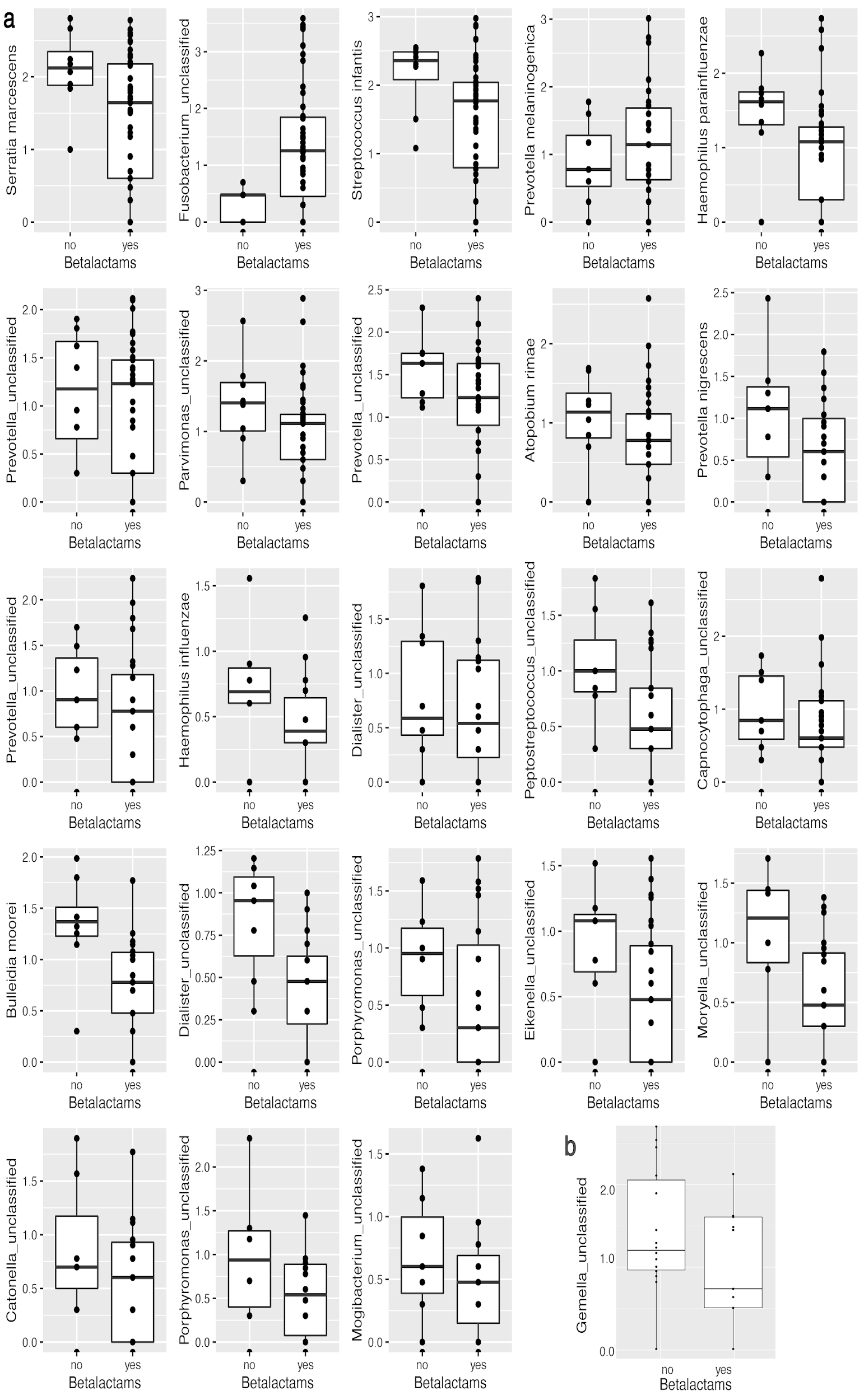


**Figure S3.** Effect of beta-lactam use on OTU abundance. Significant differences in OTU abundance based on beta-lactam use in **(a)** ICU-1 and ICU -2 **(b).** All the differences were significant according to the expected P value of Welch’s t-test and the expected P value of the Wilcoxon rank test.


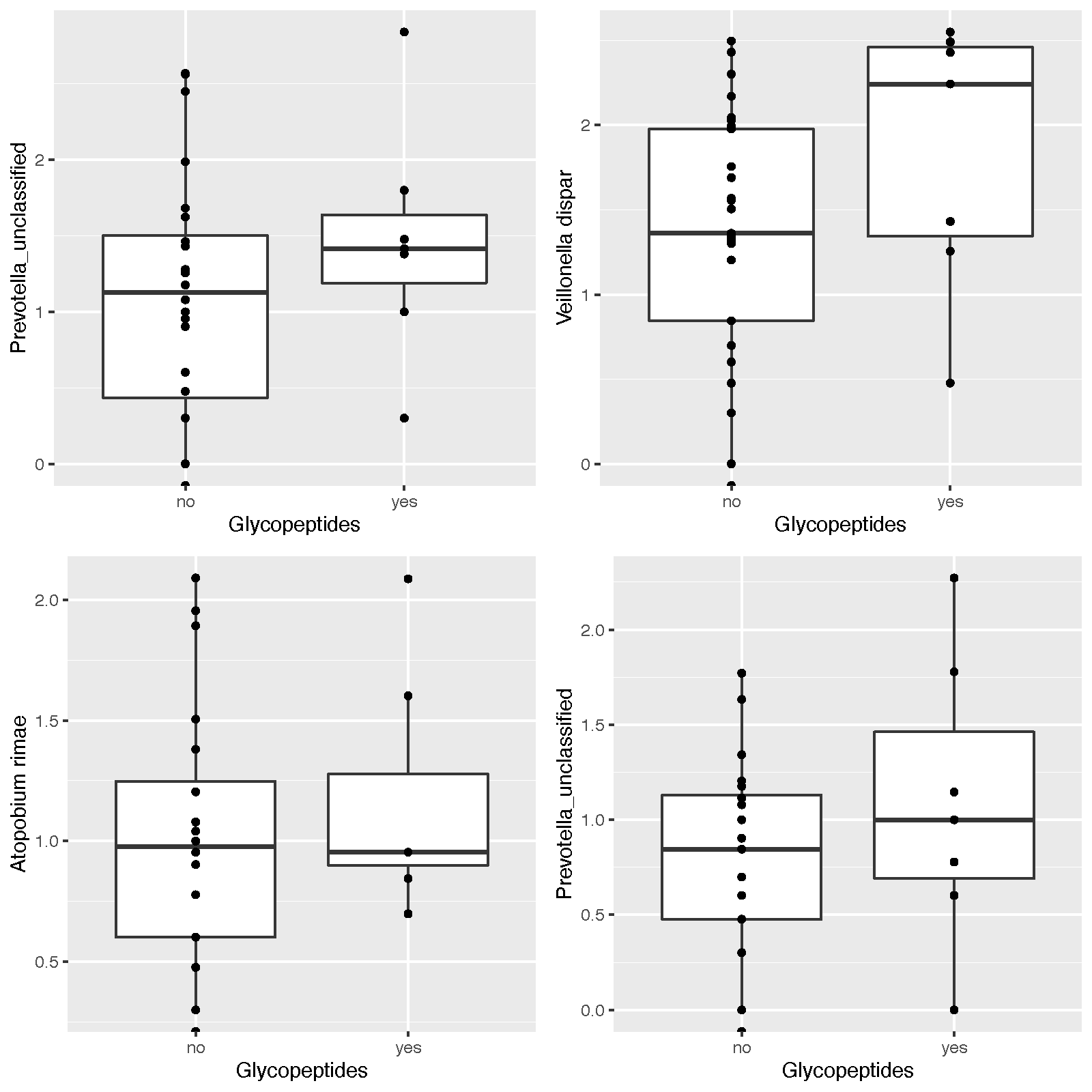


**Figure S4.** Effect of glycopeptide use on OTU abundance. The use of glycopeptides resulted in significantly different abundances of  4 OTUs in ICU-2 **.** All the differences were significant according to the expected P value of Welch’s t-test and the expected P value of the Wilcoxon rank test.
